# Received Signal Strength Indicator Measurements and Simulations for Radio Frequency Identification Tag Identification and Location in Beehives

**DOI:** 10.3390/s25113372

**Published:** 2025-05-27

**Authors:** José Lorenzo-López, Leandro Juan-Llácer

**Affiliations:** Department of Information and Communications Technology, Universidad Politécnica de Cartagena, Antiguo Cuartel de Antiguones, 30202 Cartagena, Spain; jose.lorenzo@upct.es

**Keywords:** RFID, beehive, simulation, modelling, propagation, measurements

## Abstract

The last few years have seen the introduction of new technologies in beekeeping, including RFID. Using readers and miniaturized tags, RFID systems work in the UHF frequency band, allowing reading distances to reach tens of centimeters. This work analyzes the propagation inside a full beehive, composed of 10 frames supported by a wooden structure. Each frame contains a layer of beeswax supported by metallic wires. The methodology employed involves measuring Received Signal Strength Indicator (RSSI) values and simulating the environment using CST Studio. The results show that tags located along the frame’s wires have more coverage than tags in the center positions, revealing coupling of the microtag antenna with the wire. Furthermore, obtaining coverage through simulations represents a more restrictive approach than through measurements. Frame selectivity is also observed, as most of the coverage is found within the three frames closest to the reader antenna. This result shows that RFID systems can find application in the identification and location of the queen bee in a hive.

## 1. Introduction

Bees have had a very close relationship with humans for millennia. Not only do they play a crucial role in pollination, but they also contribute to biodiversity and agricultural productivity [1,2].

However, their populations have declined in recent decades due to multiple factors including habitat loss, invasive species, emerging diseases, pesticide use, and climate change [3]. This poses a threat to global food production and ecosystem balance, highlighting the need for innovative solutions to monitor and protect these insects.

To this end, researchers and beekeepers are turning to information technology, introducing increasingly advanced monitoring systems into apiculture to facilitate hive management, improve productivity, and provide critical information on colony health and environmental interactions. One of these emerging technologies is Radio Frequency Identification (RFID) [4,5].

RFID systems comprise a reader and a tag, wherein a signal is transmitted from the former to the latter. This signal is then backscattered by the tag’s data chip, modulating it in such a way that the reader can identify it when it is within reading range [6]. RFID systems are categorized by their operating frequency band, with different frequency ranges offering different capabilities regarding reading distance and data transfer rates. RFID systems in the Ultra High Frequency (UHF) band are particularly promising as they can achieve reading distances up to tens of meters [7]. Recent advancements in antenna miniaturization permit the fabrication of smaller tags that can be attached to insects, including bees, by placing them on their thorax. This enables the study of insect behavior and monitoring systems.

Insect monitoring using RFID in the UHF band was explored intensively in [8], and other studies focused on its application in apiculture [9]. Reading distances in these studies often reach tens of centimeters [10], which is sufficient for practical applications such as bee counting systems [11] using reading antennas placed at the entrance and exit of the beehive.

Traditionally, identifying the queen bee within the hive is identified visually, i.e., by removing each frame one by one and searching for the queen—marked by a drop of paint on its thorax—among hundreds of bees. This process is time-consuming and can put the queen bee at risk due to the constant manipulation of frames. In [12], a compact 868 MHz RFID-based antenna (dimensions 3.09 × 2.61 × 0.25 mm) was developed for operation with a transponder. This allowed for the fabrication of a microtag suitable to be placed on a queen bee’s thorax. The measurements provided in [13] showed that this microtag provides sufficient range for identifying the queen bee from the exterior of a hive.

Most approaches to RFID system design are based on the simple radar equation assuming targets located in the free space and in the far field of the transmitter/receiver antennas. The complexity of real scenarios has led to the proposal of models based on transfer impedance [14], the impedance matrix [15], or S parameters [16], which consider the coupling between reader and tag antennas without any limiting assumptions on the propagation environment. The authors of [14] focused on two scenarios—free space and two-ray (direct and reflected) scenarios—for practical antenna tag design. The authors of [15] evaluated the RSSI through post-processing of the impedance matrix obtained with simulations in CST Studio (Dassault Systèmes, Vélizy-Villacoublay, France) to analyze the propagation in a drawer scenario for RFID tag classification in smart storage systems. Also, the authors of [16] used the post-processing of the S-parameters obtained from simulations in CST Studio to analyze propagation in a beehive’s honeycomb frame for the RFID of a queen bee. Our analysis exploits the fact that the most restrictive link is the downlink (reader to tag) [17].

Using simulations obtained from CST Studio, this paper analyzes propagation in a beehive when the antenna reader is located outside the hive and the microtag is located inside. The simulations are analyzed together with RSSI measurements. The objective of this work is to study the possibility of using an RFID system in the UHF band to identify and locate (at the frame level) the queen bee inside of a beehive, in such a way that it facilitates the task to the beekeeper, in a non-invasive way to the colony. This may be accomplished by moving the reader antenna along the lateral side of the hive until the tag is read.

This paper is organized as follows. The propagation environment and the measurement system are introduced in Section 2. The CST modelling approach of the scenario is described in Section 3. The methodology is presented in Section 4. The results are shown and discussed in Section 5. Finally, concluding remarks are given in Section 6.

## 2. Environment

**Figure 1:**
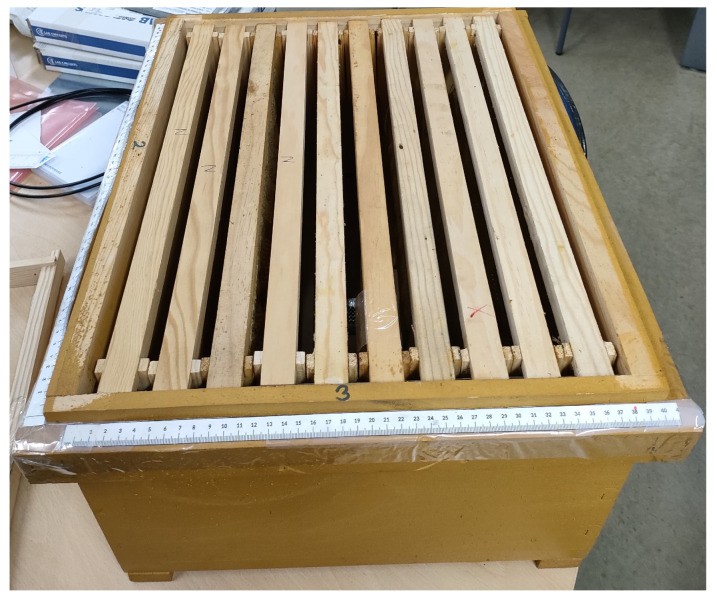
Overview of the full hive considered in this work.

This study considers a beehive as the propagation environment, specifically a Dadant beehive, which consists of a wooden box that supports 10 honeycomb frames. The dimensions of the whole hive are 51 × 42.5 × 31 cm. Figure 1 gives a general overview of the beehive, showing that all frames can be viewed from the top.

**Figure 2:**
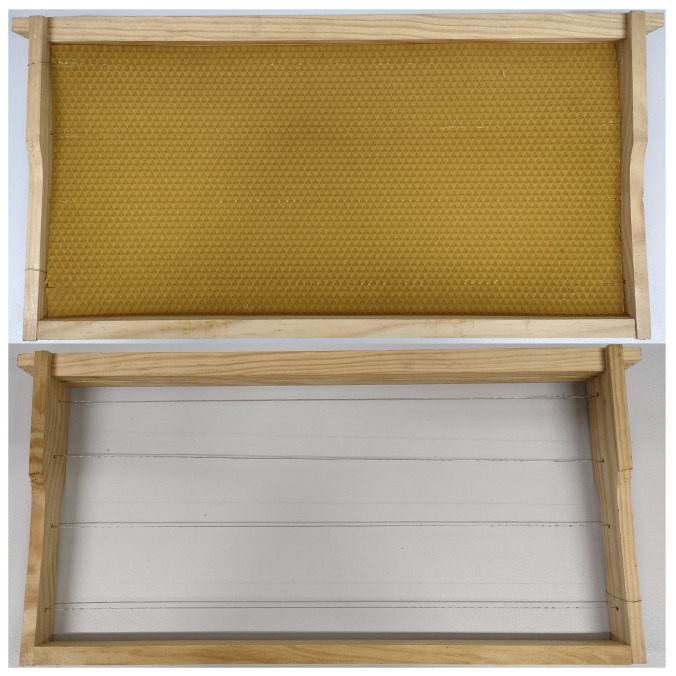
Honeycomb frame of the hive considered with beeswax (**top**) and no beeswax (**bottom**).

Each of the frames is composed of a wooden structure with outer dimensions of 44.5 × 23.5 cm, a thin layer of beeswax that serves as a guide for the bees to build their cells, and two metal wires running throughout the wooden structure to hold the beeswax layer. When beekeepers excite these wires with an electrical current, they serve as heaters, melting the beeswax and fixing it to the wires. A lateral view of a honeycomb frame is presented in Figure 2, with one frame with and one frame without beeswax to show the metal wires inside the structure.

The wires significantly influence electromagnetic propagation as they act as metal conductors, becoming subjects of magnetic coupling with external fields.

### 2.1. Electrical Permittivity of Beeswax

To develop a more realistic model of the problem, it is essential to characterize the electrical properties of all materials comprising the hive. While the permittivity of beehive materials such as wood and metal wire is well documented due to these materials’ widespread industrial applications, there is limited knowledge of the permittivity of beeswax, particularly in the UHF frequency band. Moreover, although some studies have investigated the permittivity of honey [18], recent work specifically characterizing beeswax in this frequency range is lacking. Therefore, it is necessary to analyze this property.

**Figure 3:**
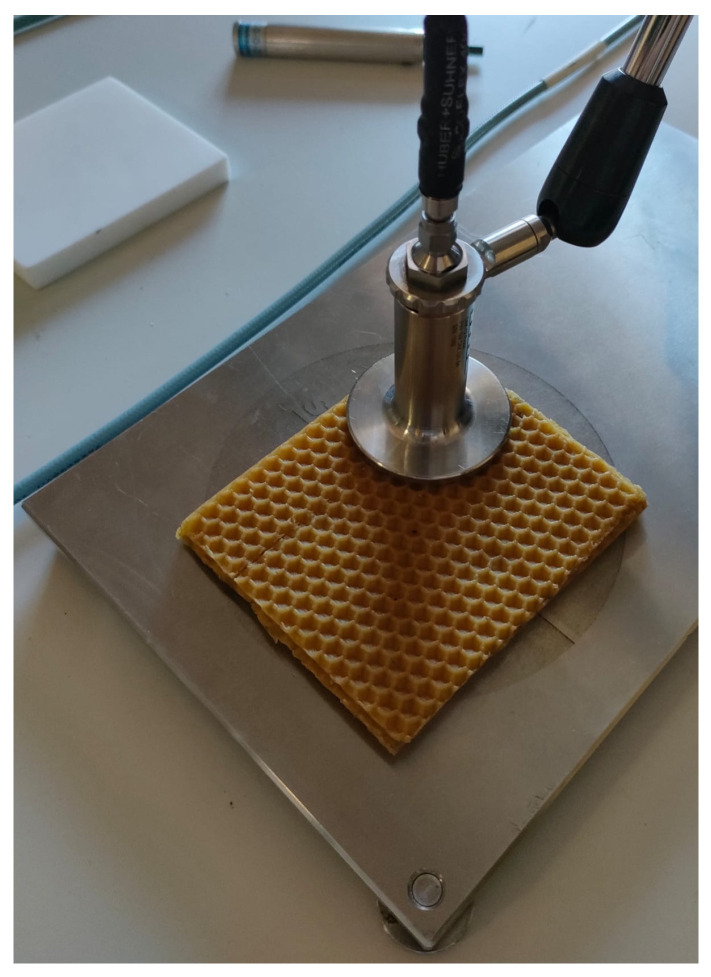
Photo illustrating the permittivity measurement procedure using the DAK probe.

The device used to perform the dielectric measurement was a dielectric probe kit DAK from SPEAG connected to a Vector Network Analyzer (VNA), as described in a similar dielectric study [19]. A sample measurement is given in Figure 3.

**Figure 4:**
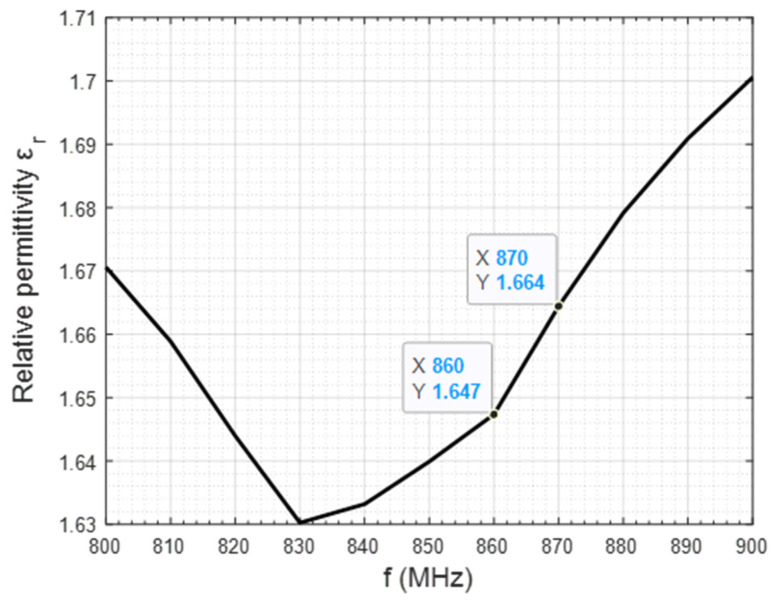
Relative permittivity of beeswax as a function of the frequency (from 800 to 900 MHz).

Permittivity measurements were performed in nine different positions to minimize probe placement-induced error. The average value obtained from these nine results is shown in Figure 4 in a frequency range from 800 to 900 MHz.

As our work frequency is fixed at 868 MHz, we take the closest permittivity value, located at 860 and 870 MHz, which is 1.65, rounded to the second decimal point. This value is then introduced into CST Studio to define the electrical permittivity of the material comprising the beeswax element.

### 2.2. Measuring System

The RFID system consists of a reader and a tag. The reader employed is the Speedway Revolution R420 (Impinj, Seattle, WA, USA), which supports the EU1 UHF frequency band (865–868 MHz) and offers a sensitivity of −84 dBm, with a maximum transmitted power of 31.5 dBm. It is connected to an SP11 Keonn Advantenna (Keonn Technologies, Barcelona, Spain), which has a gain of 5.1 dBi [20]. The dimensions of the antenna are 20.7 × 20.7 cm.

The label/microantenna was carefully customized considering that, on the one hand, it would be used in a biological application and, on the other hand, that, being attached to the thorax of the queen bee, the latter would suffer the least possible impact. In this sense, the following considerations were taken into account [12]:

- Small dimensions of the label to fit the queen bee’s thorax: 3.09 × 2.61 × 0.25 mm.
- Small weight of the label: 0.025 g, which roughly represents 10% of the average weight of a queen bee.
- Radiation pattern of the microantenna with a radiation null in the direction of the queen bee’s body:
- The lifespan of the tag is expected to match that of a queen bee, which typically ranges from 1.5 to 3 years, either until it naturally expires or is replaced by the beekeeper.
- UHF frequency band (specifically, 868 MHz).
- Impedance matching of the antenna and chip for maximum power transfer.

The compact antenna has been designed to operate at 868 MHz with the Murata LXMS21ACMD-218 transponder (Murata Manufacturing Co., Ltd., Kyoto, Japan) [21]. This transponder incorporates the Impinj Monza R6 microchip (Impinj Inc., Seattle, WA, USA) [22], boasting an input impedance of 13.4–j126 Ω at the operating frequency.

**Figure 5:**
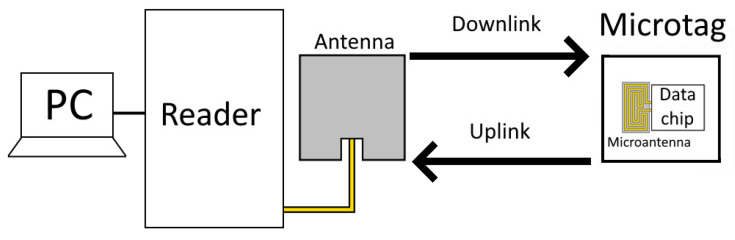
Block diagram of the measuring system.

The passive tag receives the signal provided by the reader and backscatters it, provided that the received power is above its sensitivity of −22 dBm. When the backscattered signal is returned to the reader, it displays the tag name and RSSI value if the received power is above the reader’s sensitivity of −84 dBm. A diagram of the system is shown in Figure 5.

**Figure 6:**
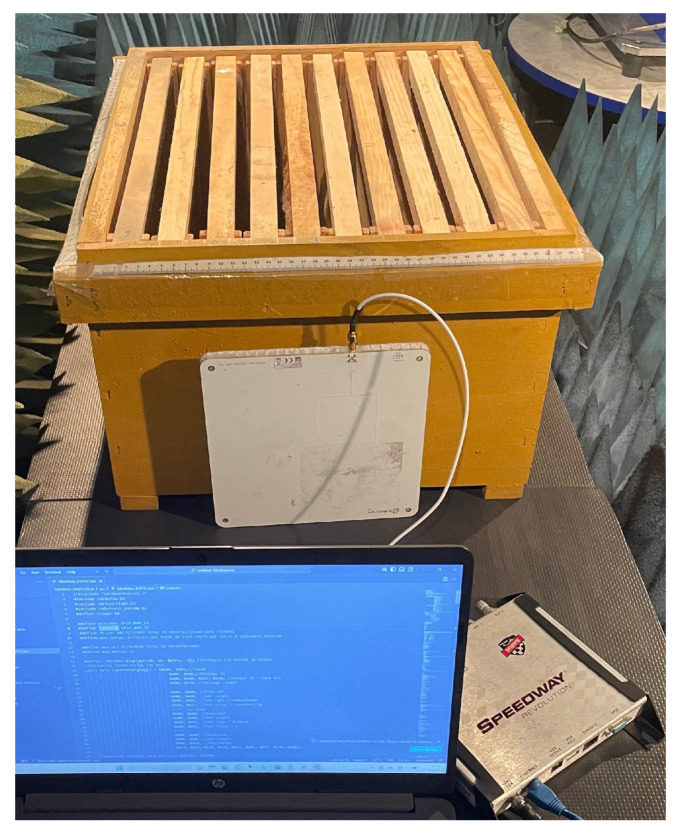
Measurement setup of the RSSI values in the beehive.

As discussed in [17], the most restrictive link in this communication is the downlink, i.e., from the reader to the tag. This will determine our measurements and result interpretations in further sections. A full view of the RFID system and the beehive is presented in Figure 6.

## 3. CST Modelling

Once all structural materials are characterized, they can be used to model a full beehive in CST Studio. The resulting model has the same physical size as the beehive, as well as the same electrical properties. Different materials were employed to construct the model, as detailed in Table 1.

**Figure 7:**
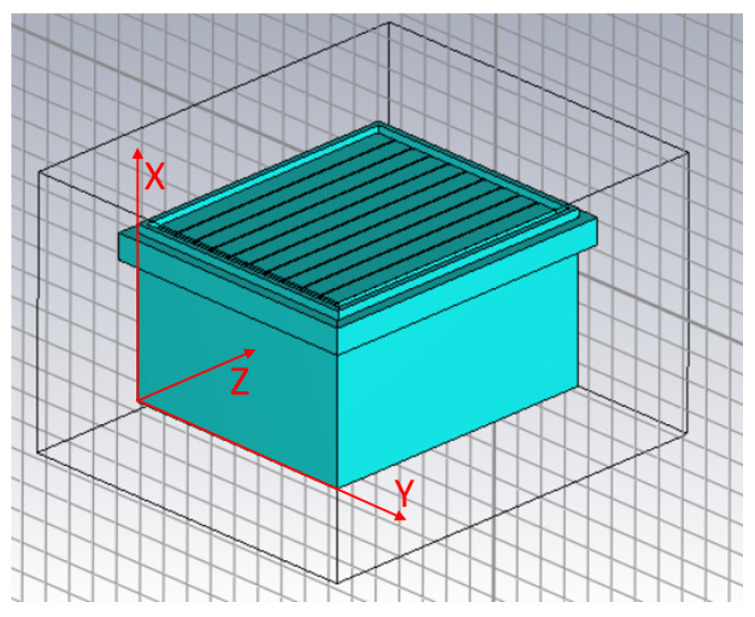
Overview of the full hive in CST Studio.

**Figure 8:**
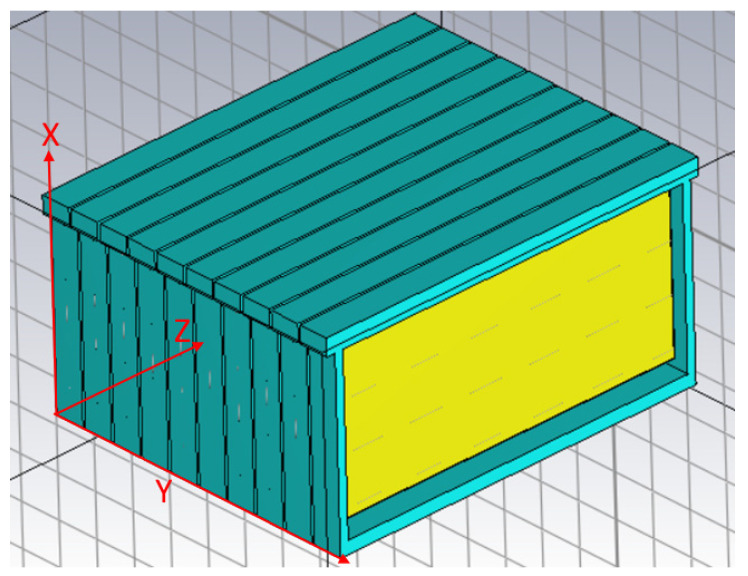
Overview of the 10 honeycomb frames composing the hive in CST Studio.

The following figures show the resulting model in the program, whereby each material is colored differently. In Figure 7, wood is blue, whereas in Figure 8, beeswax is yellow. The metal wires inside the beeswax are also worth noting.

**Figure 9:**
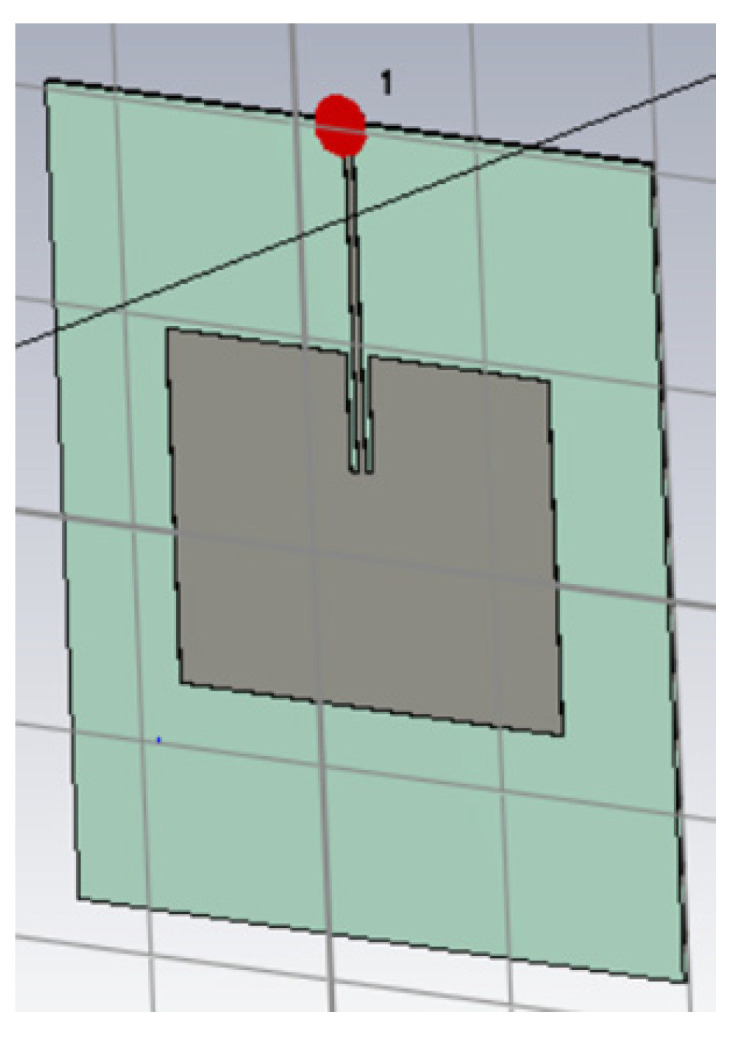
Reader antenna modeled in CST Studio.

A model of the reader antenna is presented in Figure 9. In this case, the substrate is light blue, whereas the patch antenna is gray. The working frequency is 868 MHz, obtaining a gain of approximately 5.6 dBi.

**Figure 10:**
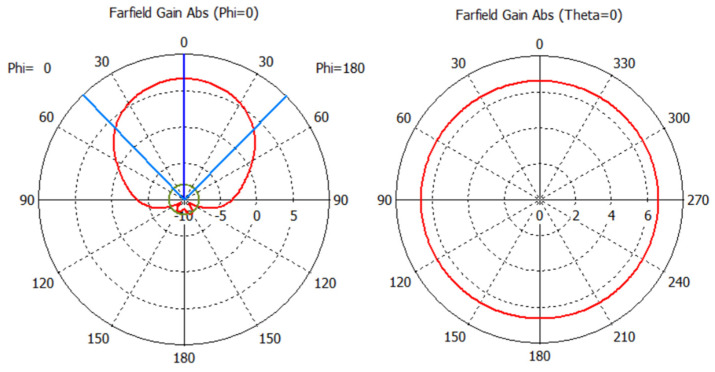
Radiation patterns of the modeled reader antenna.

Figure 10 shows the radiation patterns of the reader antenna obtained in CST Studio.

## 4. Methodology

To analyze the coverage of the beehive, a series of both theoretical simulations and real measurements were performed.

**Figure 11:**
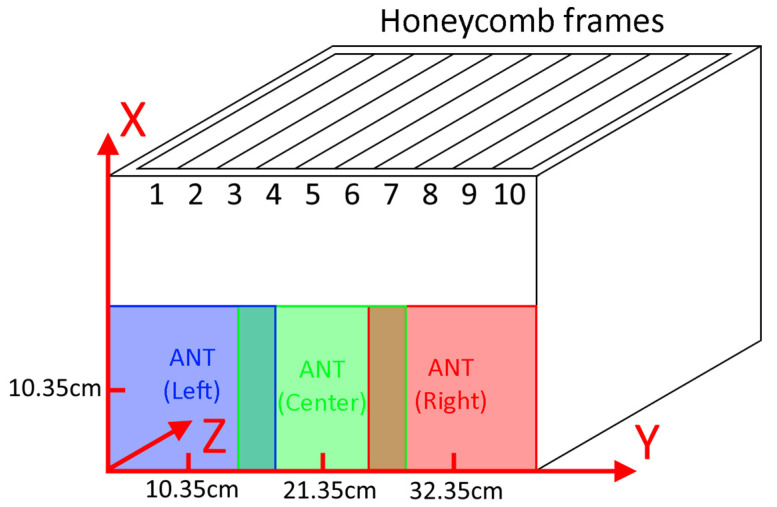
Reader antenna placements along the side of the beehive.

The reader antenna was placed over the lateral face of the hive, facing the frames perpendicularly. Three different cases were considered: antenna placed on the left side (centered on x = 10.35 cm, y = 10.35 cm), the center (x = 10.35 cm, y = 21.35 cm), and the right side (x = 10.35 cm, y = 32.35 cm). Figure 11 shows the different antenna positions.

**Figure 12:**
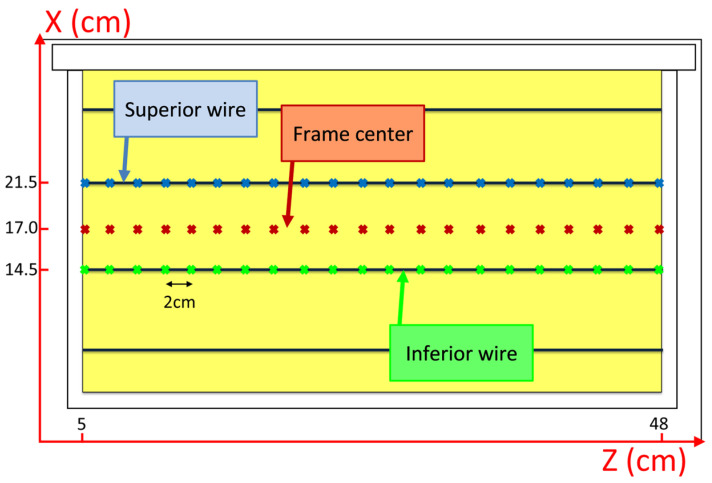
Tag measurement positions in each frame of the beehive.

For each honeycomb frame inside the beehive, a total of 22 points, separated by 2 cm, were established. Three zones (superior wire, frame center, and inferior wire) were also distinguished in each frame: over the second wire from top to bottom (x = 20.5 cm), between the second and third wires (x = 17 cm), and over the third wire (x = 14.5 cm). This produced a total of 66 points in each frame, as shown in Figure 12.

### 4.1. Theoretical Simulations

CST Studio permits the retrieval of numerous variables when it comes to simulation results. Due to our focus on the most restrictive link, i.e., the downlink, a very desirable variable to be obtained is the power received by the tag antenna. This power received at a given point can then be compared to the tag sensitivity of −22 dBm to determine whether there will be coverage at that specific point.

**Figure 13:**
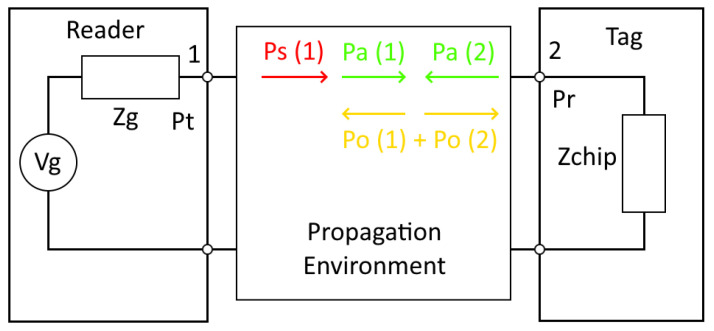
Block diagram regarding the power definition within the system in CST Studio.

In order to find the power received in the tag, we can characterize the full medium by its S-parameters, as studied in [16]. Port 1 of the system will be the reader antenna, and Port 2 the tag microchip. Our approach to the problem follows this scheme as well. The difference is that by setting Port 1 as the excitation port, we can directly obtain the power received in the tag in the downlink, by looking at CST Studio power definitions [23]. Thus, we can express the power as follows:

(1) $$P_{s}\left(W\right)=P_{a}(W)+P_{o}(W)$$

where $P_{s}$ is the power stimulated per port, $P_{a}$ is the power accepted per port, and $P_{o}$ is the outgoing power from all ports. Figure 13 presents a general diagram.

By setting Port 1 connected to the reader antenna and Port 2 connected to the tag antenna, the power stimulated is 0.5 W in Port 1 (obtained from an input power of 1 W) and 0 W in Port 2, as the tag is considered the end point of the downlink. When looking at the preliminary results, we discover that the power accepted in Port 2 is shown as a negative value. Substituting (1) for Port 2, we find

(2) $$P_{a}\left(W\right)=-P_{o}(W)$$

Hence, the power accepted is interpreted as the outgoing power from Port 1 into Port 2, i.e., the power received at the tag data chip. As the reference power is 1 W for Port 1, we can obtain the expression to calculate the received power in the tag:

(3) $$P_{r}(\mathrm{dBm})=P_{t}\left(dBm\right)+10\cdot log10(P_{a}(W)/P_{ref}(W))$$

where $P_{r}$ is the power received in the tag, $P_{t}$ is the power transmitted from the reader, and $P_{a}$ is the power accepted in the tag port. $P_{ref}$ is the reference power in the ports, which is 1 W. Then, taking into account that $P_{t}$ is 31.5 dBm, the power received in the tag is

(4) $$P_{r}(\mathrm{dBm})=31.5dBm+10\cdot log10(P_{a}(W)/1W)$$

As a full simulation had to be performed to obtain the power received in the tag port for a single point, characterizing the 660 points of the total beehive implied a sweep simulation of 660 iterations. This process would have taken considerable computational time and required a large resource allocation. In the end, this approach was discarded.

**Figure 14:**
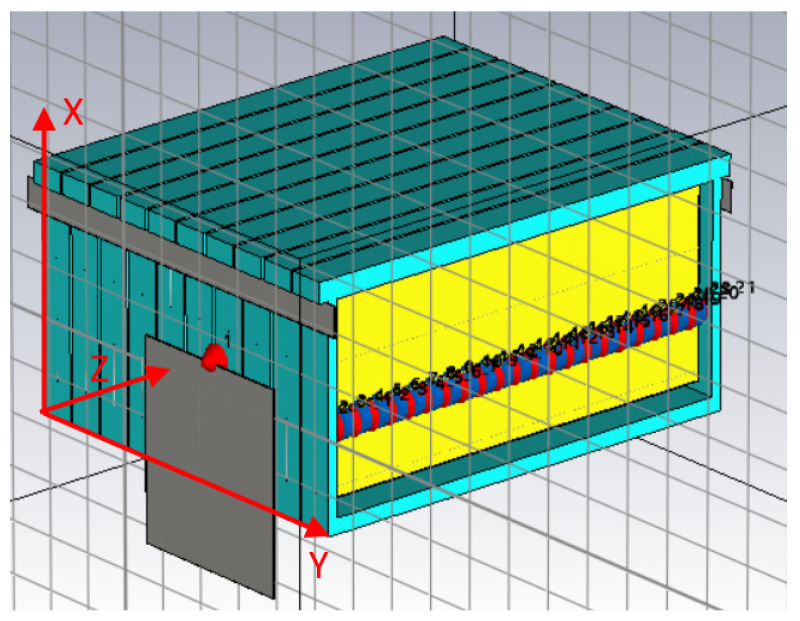
The 22 positions of the tags throughout a frame of the hive in CST Studio.

The problem was solved by setting 22 tags, covering the 22 points of a single zone in a honeycomb frame. A view of the process is given in Figure 14.

This approach increased the simulation time of a single iteration but greatly reduced the overall time needed to process the 660 points.

Another issue when setting the simulations was the meshing passes. A higher number of passes provides more precise results while increasing the computational time. To determine this number, a series of simulations was carried out in the same scenario, while only changing the meshing passes. Three cases were considered: eight passes, four passes, and three passes. The best case, i.e., that with the most precise result for power received, was eight passes. Its computational time was significantly higher than the other two cases, reaching over 4 h of simulation. Table 2 shows the average error of received power in the tag with respect to the eight-pass case.

As can be seen, the error in the four-pass case is significantly inferior to that in the three-pass case. It is also worth noting that in every case, the least number of passes returned to a lower received power in the tag. This means that a small error is tolerable as it can provide a more restrictive solution, so the four-pass meshing was used for the simulations.

### 4.2. RSSI Measurements

To determine the system’s coverage, Relative Signal Strength Indicator (RSSI) measurements were carried out. RSSI measures the quality of the communication channel and its value, in dBm. When an RSSI value is measured, enough power has been supplied to the tag to generate a signal that is sent back to the receiver. As commented upon in [17], the most restrictive link in an RFID communication is the downlink. In other words, when an RSSI value is obtained at the reader, we know that $P_{r}>S$, where $P_{r}$ is the received power in the tag obtained in (4) and S is the sensitivity of the tag. In our study, this sensitivity is −22 dBm.

This relationship is fundamental to comparing results between simulations and RSSI measurements. Essentially, when the simulation provides a value of power in the tag over −22 dBm, we can check in the measurements whether an RSSI value was found for the same tag position.

The measurements were performed inside an anechoic chamber using the system described in Section 2.

## 5. Results

This section compares the theoretical simulations with the RSSI measurements. First, the preliminary RSSI measurements are presented. Then, the full hive RSSI values and theoretical results are compared. Finally, a coverage map is presented.

### 5.1. RSSI Measurements

**Figure 15:**
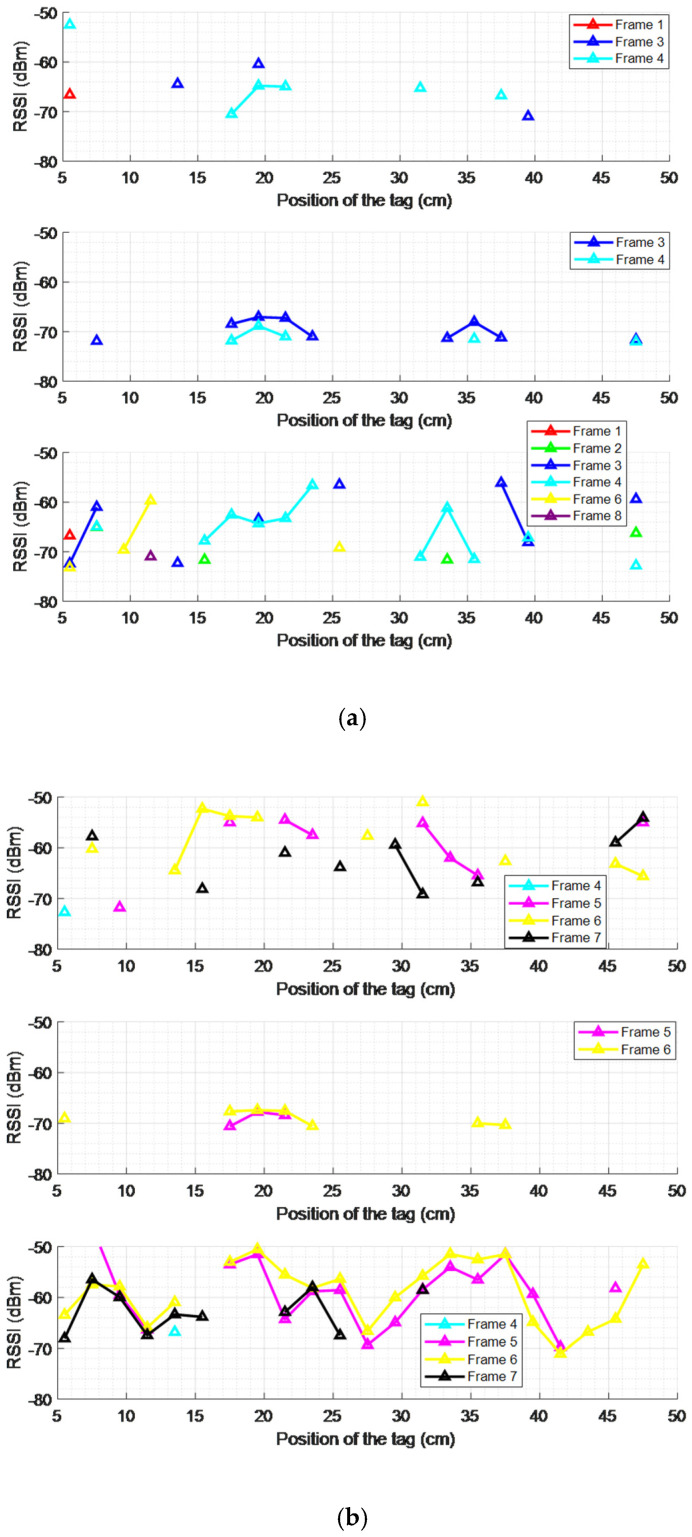

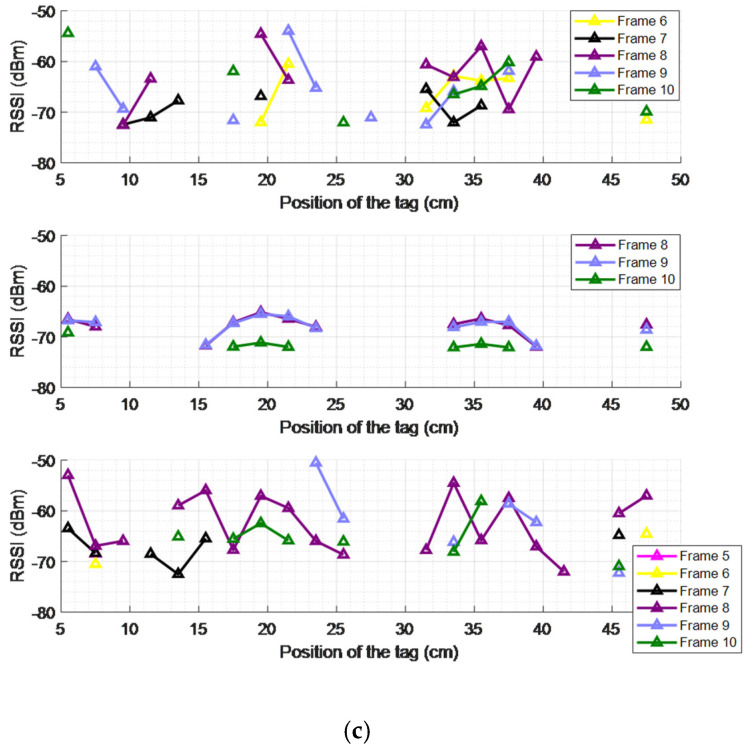
RSSI measurements versus the position of the tag for different frames, considering the superior wire (top), the frame center (middle), and the inferior wire (bottom) for the left (**a**), center (**b**), and right (**c**) reader antenna placements.

A preliminary campaign of measurements was performed over the different honeycomb frame zones defined above (see Figure 12), with respect to the antenna placement. Figure 15a shows the RSSI values for the left antenna placement, Figure 15b for the centered case, and Figure 15c for the right antenna position.

As can be seen, the tags located along the wires (bottom or top) have more coverage than the tags in the center positions, revealing coupling of the microtag antenna with the wire. Furthermore, the tags located on the bottom wire have more coverage positions than those on the top wire because the direction of the reader antenna’s maximum radiation is closer to the bottom wire than the top. Table 3 compiles the total number of points where there was an RSSI value for each tag position and antenna placement.

Further sections only consider the inferior wire for the analysis of RSSI measurements and simulations, lowering the computational time required as fewer points have to be simulated.

### 5.2. RSSI Values and Power Received

This subsection covers the measurements performed in a real beehive, as well as the theoretical values obtained in the CST Studio simulations of the full environment. In the first case, RSSI is discussed, whereas in the second case, the simulated power received in the tag is the result value. Importantly, even though they have a direct relationship, these values cannot be compared directly, as explained in Section 4.

Over the next figures, the RSSI values are represented along with the simulated power received in the tag in CST Studio. Each figure represents a different antenna configuration, and only the inferior wires are considered for each honeycomb frame. Only frames with both theoretical and measured coverage are displayed.

**Figure 16:**
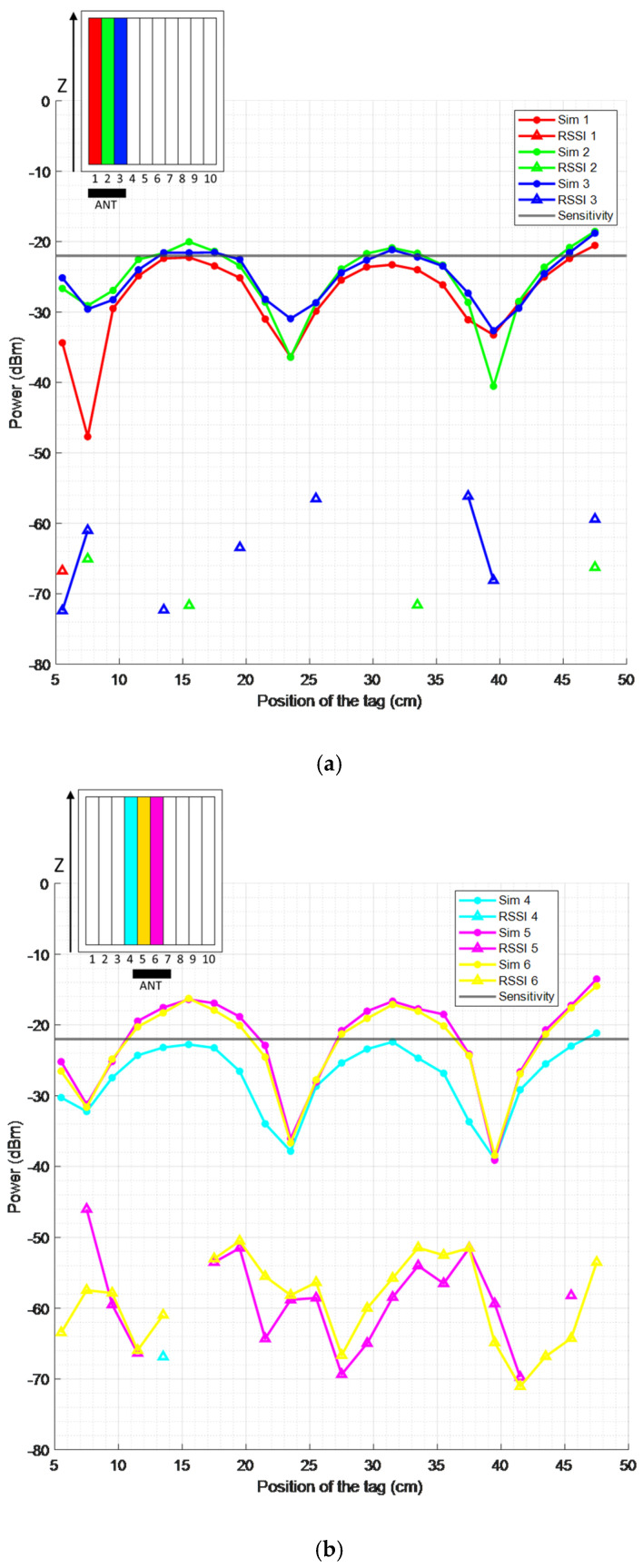

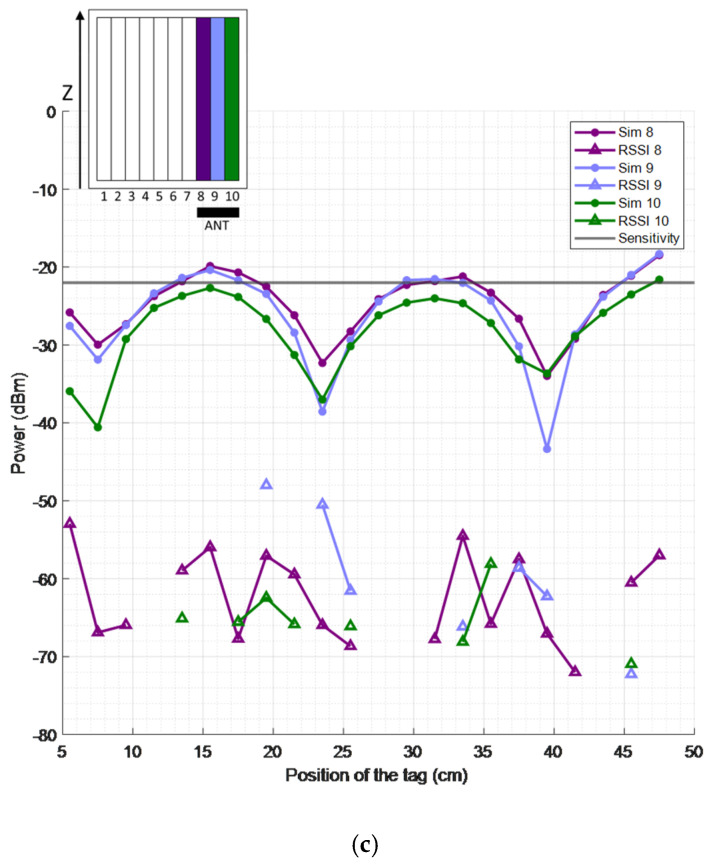
RSSI measurements and simulated received power versus the position of the tag, considering different frames, for the left (**a**), center (**b**), and right (**c**) reader antenna placements.

We can observe that the RSSI values are between −75 and −55 dBm in Figure 16a, −45 to −70 dBm in Figure 16b, and −50 to −70 dBm in Figure 16c. These values fluctuate and do not follow a shape comparable to the theoretical power received in the tag. This result was also observed in [16], which analyzed only a single honeycomb frame. However, some RSSI values can be seen in zones where the received power is maximum, even when it is not above the tag sensitivity. Similarly, some zones have no RSSI values in any case, corresponding to points where the theoretical power received is at a minimum.

Overall, the theoretical coverage is much lower than that measured, as very few points of received power are above the tag sensitivity compared to the number of points where an RSSI value is found.

Furthermore, as seen throughout Figure 16, there is a certain selectivity between frames, as only three frames have both theoretical and measured coverage per antenna placement. This behavior, also observed in [15] for tag classification in a smart storage system, is advantageous for locating the queen bee, as the fewer frames covered for a specific antenna placement, the less uncertainty regarding the frame in which the queen bee is located.

This is also corroborated by looking at the frame numbers displayed. We can see in Figure 16a that when the antenna is in the left position, only frames 1, 2, and 3 have both theoretical and measured coverage. This behavior is symmetrical with respect to Figure 16c, corresponding to the right antenna placement, where the rightmost frames are displayed: 8, 9, and 10. We can also observe in Figure 16b that for the centered antenna placement, the frames shown are 4, 5, and 6, which do not follow a perfect symmetry but are all different from the other cases.

Another noticeable result is the periodicity in the behavior of the power received, which was also observed in [16]. As can be seen, the local minima present in the power received on the label are spaced approximately 16 cm apart. This distance can be related to half of the wavelength at the operating frequency, which is 34.56 cm at 868 MHz. Considering that the effective relative permittivity of the medium (including air and beeswax) will be higher than 1, it is coherent to assume that half of the resulting wavelength will be close to the 16 cm observed. This behavior is explained by the presence of reflections in the hive environment, provoking standing waves that minimize the power received in certain points.

### 5.3. Coverage

Previous results can help understand the relationship between the power received and the RSSI values obtained. However, it is also important to compare the number of points where there is theoretical and measured coverage. This property is displayed as a two-colored map. The blue points correspond to no coverage, and the yellow points correspond to a coverage point. If looking at theoretical simulations, this means that above that point, the power received is higher than the tag sensitivity; however, in the measurements, it means that an RSSI value was obtained for that point. Over the vertical axis, the distance over the frame is displayed, and in the horizontal axis, the frame number is shown.

**Figure 17:**
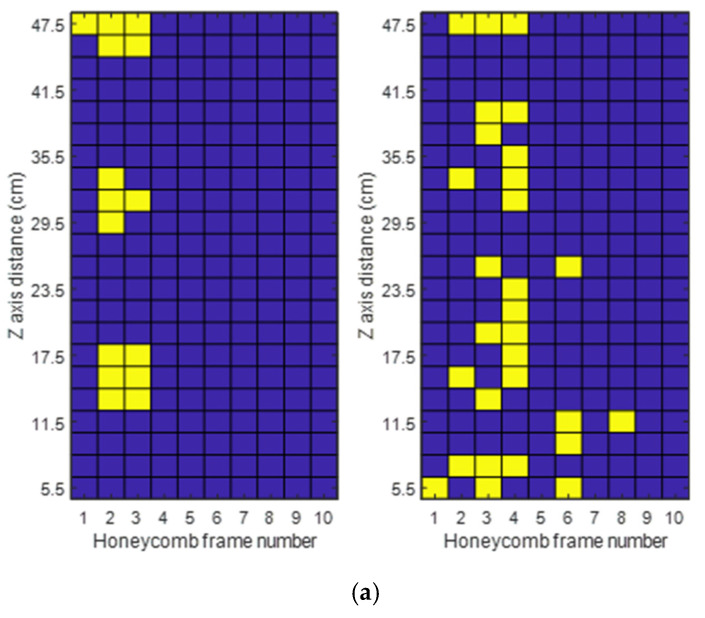

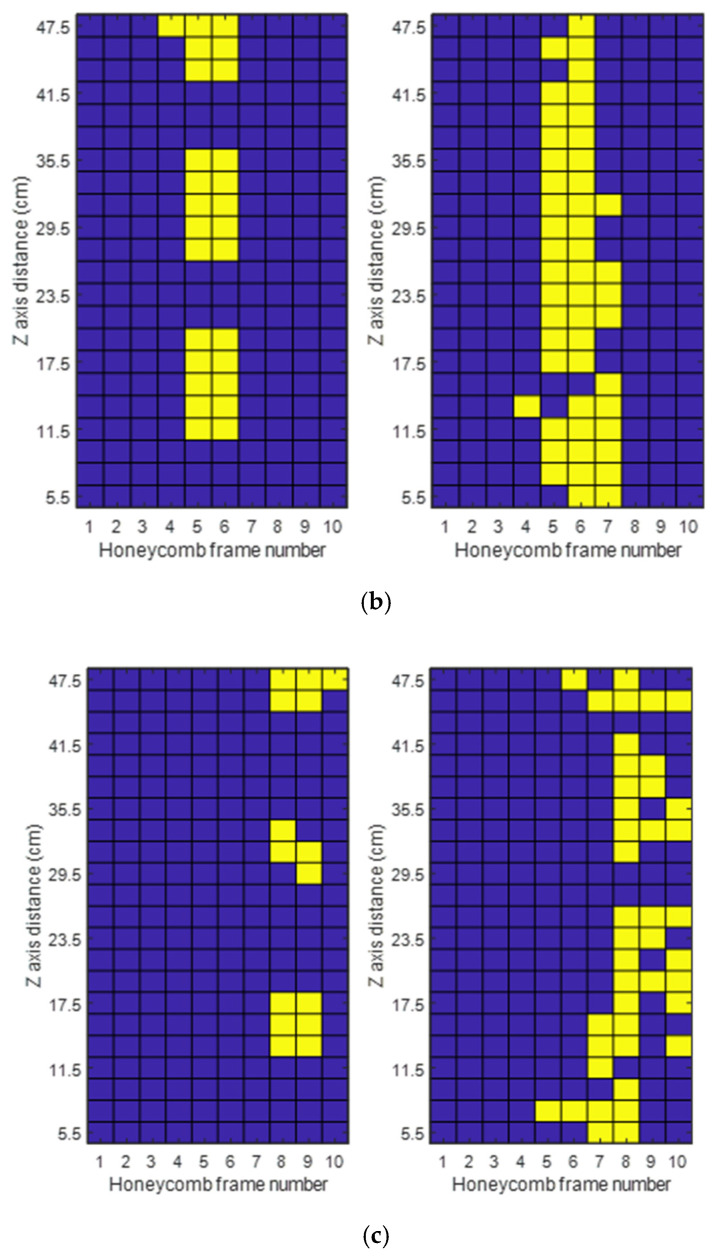
Simulated (left) and measured (right) coverage of the full hive for the left (**a**), center (**b**), and right (**c**) antenna reader placements.

Considering Figure 17a, we find that the simulation returns three different zones of coverage, covering mainly frames 2 and 3. The measured coverage provides a scattered pattern, covering mainly frames 3 and 4. However, more points of coverage are found throughout the hive, reaching a single point of coverage in frame 8.

Figure 17b, corresponding to the centered position, is the one where the simulation coverage is highest, offering three zones, similar to the previous case for frames 5 and 6. The measured coverage is almost full in frames 5 and 6, and almost half of frame 7 is also covered.

Finally, Figure 17c, the right antenna placement, provides the lowest simulated coverage, which is also spread among three zones, but is present in frames 8 and 9. The measured coverage is mainly focused on frame 8, but almost half of frames 7 and 9 are also covered, as well as a significant part of frame 10.

In order to better quantify the simulated and measured coverages, the coverage as a percentage is compiled in Table 4 for each frame, depending on the antenna placement for both simulations and measurements.

We can observe that higher coverage is obtained in the measurements rather than in the simulations. This means that the CST simulations provide a more restrictive model of coverage compared to the real environment, which is a desirable result.

As seen earlier, the best case is the centered antenna placement, where frames 5 and 6 have coverage in 59.1% of the points for the simulations and 77.3% and 95.5%, respectively, in the measurements. Overall, the selectivity between frames observed in previous subsections is shown in the table, as the frames with the most coverage are located in the same place as the antenna. For the left antenna placement, frames 1, 2, and 3 are covered; for the center placement, frames 5 and 6 (arguably 4 and 7 could also be included); and for the right antenna placement, frames 8, 9, and 10.

## 6. Conclusions

Firstly, tags located along the wires (bottom or top) have more coverage than tags in the center positions, revealing coupling of the microtag antenna with the wire.

Secondly, a symmetrical behavior is observed in the coverage of the simulated hive, when the reader antenna is placed at the left and right positions. However, this symmetry is much less apparent in the real hive. This could be caused by certain elements that were not taken into account in the simulations due to their complexity, such as bends in the wires, uneven separation between frames, deviations of the wires with respect to the Z axis, etc.

As observed in [16], a periodic behavior is shown in the power received in the tag. This is caused by the formation of standing waves, which can make determining the location of the queen bee at certain points difficult.

Finally, coverage maps obtained in the CST simulations provide a more restrictive model compared to the RSSI measurements, as fewer points with coverage were obtained. Despite this, both the measurements and simulation results prove that there is selectivity between frames, as the majority of the coverage points for any antenna placement were found within the two or three closest frames. This result shows that RFID systems can find application in the identification and location of the queen bee in a hive, as ambiguity in the frames needed to be inspected by the beekeeper are lowered from 10 to a maximum of 3. This saves time and lowers the overall stress induced and risk of killing the bees accidentally. Furthermore, if the location of the queen bee is not needed at a certain time but its tag’s parameters are it is possible to read the tag from the outside of the beehive. This allows the beekeeper to obtain the insect’s parameters without having to open the hive, saving time as well.

As future work, both a theoretical analysis and its corresponding measurement campaign will be performed for the case of an environment in which bees and substances typical of a beehive are present, resulting in a logical complement to the results shown in this preliminary work.

## Figures and Tables

**Table 1:** CST Studio materials employed.

Name	Element	Property
PEC	Hive metal plates, reader antenna	σ=∞ S/m
Beeswax	Honeycomb frame	$\varepsilon_{r}=1.65$
Steel-1008	Frame wires	σ=7.69 MS/m
Wood	Frame and hive structure	$\varepsilon_{r}=2.014135$ −j0.3424452
Copper (pure)	Tag antenna	σ=59.6 MS/m
FR-4	Tag antenna substrate	$\varepsilon_{r}=4$ tanδ=0.02
FR-4	Reader antenna substrate	$\varepsilon_{r}=4.3$

**Table 2:** Average error with respect to the best case.

Number of Passes	Computational Time	Average Error (dB)	Maximum Error (dB)
3	Less than 30 min	5.11	6.54
4	Less than 1 h	1.57	2.62

**Table 3:** Comparison of the number of detected points between the tag positions.

Tag Positioning	Antenna Placement		
	Left	Center	Right
Superior wire	10	28	39
Frame center	14	10	32
Inferior wire	29	49	42

**Table 4:** Percentage (%) of coverage for each frame depending on antenna placement for the simulations and RSSI measurements.

Honeycomb Frame	Antenna Placement (Simulations)			Antenna Placement (Measurements)		
	Left	Center	Right	Left	Center	Right
Frame 1	4.5	0	0	4.5	0	0
Frame 2	36.4	0	0	18.2	0	0
Frame 3	27.3	0	0	36.4	0	0
Frame 4	0	4.5	0	50	4.5	0
Frame 5	0	59.1	0	0	77.3	4.5
Frame 6	0	59.1	0	18.2	95.5	9.1
Frame 7	0	0	0	0	45.5	27.3
Frame 8	0	0	31.8	4.5	0	81.8
Frame 9	0	0	31.8	0	0	31.8
Frame 10	0	0	4.5	0	0	36.4

## Data Availability

The raw data supporting the conclusions of this article will be made available by the authors on request.
